# Porous silicon nanocrystals in a silica aerogel matrix

**DOI:** 10.1186/1556-276X-7-397

**Published:** 2012-07-17

**Authors:** Jamaree Amonkosolpan, Daniel Wolverson, Bernhard Goller, Sergej Polisski, Dmitry Kovalev, Matthew Rollings, Michael D W Grogan, Timothy A Birks

**Affiliations:** 1 Department of Physics, University of Bath, Claverton Down, Bath, BA2 7AY, UK; 2 Infineon Technologies AG, Siemensstrasse 2, Villach, 9500, Austria; 3 Department of Energy & Hydrocarbon Chemistry, Graduate School of Engineering, Kyoto University, Nishikyo-ku, Kyoto, 615-8510, Japan; 4 Department of Electrical and Computer Engineering, Boston University, Boston, MA, 02215, USA

**Keywords:** porous silicon, nanoparticle, luminescence, Raman, silica aerogel, oxygen, energy transfer, 78.66.Jg, 78.67.-n, 78.67.Bf

## Abstract

Silicon nanoparticles of three types (oxide-terminated silicon nanospheres, micron-sized hydrogen-terminated porous silicon grains and micron-size oxide-terminated porous silicon grains) were incorporated into silica aerogels at the gel preparation stage. Samples with a wide range of concentrations were prepared, resulting in aerogels that were translucent (but weakly coloured) through to completely opaque for visible light over sample thicknesses of several millimetres. The photoluminescence of these composite materials and of silica aerogel without silicon inclusions was studied in vacuum and in the presence of molecular oxygen in order to determine whether there is any evidence for non-radiative energy transfer from the silicon triplet exciton state to molecular oxygen adsorbed at the silicon surface. No sensitivity to oxygen was observed from the nanoparticles which had partially H-terminated surfaces before incorporation, and so we conclude that the silicon surface has become substantially oxidised. Finally, the FTIR and Raman scattering spectra of the composites were studied in order to establish the presence of crystalline silicon; by taking the ratio of intensities of the silicon and aerogel Raman bands, we were able to obtain a quantitative measure of the silicon nanoparticle concentration independent of the degree of optical attenuation.

## Background

Silica aerogels are rigid, highly meso-porous structures with pores on the scale of about 10 to 50 nm with a range of extreme properties: these include low refractive index (1.02 to 1.05 compared to 1.46 for bulk silica in the green spectral region), low density (greater than 90% air by volume), high optical transparency and low thermal conductivity. They have recently been demonstrated to provide a gas-permeable support for many types of nanoscale materials (metal nanoparticles, dye molecules, or semiconductor nanoparticles; see, for example, [1-4]) and, thus, are of potential interest for applications such as catalysis [5], sensing, photochemistry and coherent anti-Stokes Raman [6]. Aerogels are readily prepared from the liquid phase in arbitrary shapes and large volumes and so provide the possibility of long path lengths (of, quite easily, several centimetres) for light interacting with any embedded, optically active nanoparticles.

Porous silicon (PSi) grains may be formed by very diverse means: these include the electrochemical etching of bulk single-crystal substrates [7] followed by pulverisation of the etched layer or, as for all the porous particles here, the stain etching of macroscopic silicon powder grains [8]. Depending on the details of the etching process, the etched pores can be selected to be in the micro-, meso- or macro-porous range (approximately 2, 2 to 50, and > 50 nm respectively). By these routes, very large surface area to volume ratios may be obtained. The porous silicon surface can be prepared with hydride (Si-H) or oxide (Si-O-Si) termination, or thermally hydrosilylated to facilitate anchoring of a wide range organic molecules [9]. The presence of quantum-scale crystalline silicon particles leads to optical emission bands which can be tuned through the visible and near-IR spectral range and which can easily dominate the optical response of the composite material [9,10]. Furthermore, energy transfer via excitons within the porous silicon nanoparticles (PSi NPs) to chemical species adsorbed on (or bonded to) the NP surface has been demonstrated for a number of chemical species [11-13]; this process gives the potential for a connection between optical excitation of the composite and gas-phase chemistry within the silica pores.

However, only a few studies have been carried out on aerogel-silicon nanoparticle composites, and the main focus has been on Si nanoparticles (rather than porosified Si grains) aimed at optoelectronic applications [14] though some composites have been produced by pressing pellets of mixed PSi and aerogel [15]. Some basic questions must be answered before applications of such composites can be addressed. Is it possible to prepare a silica aerogel with embedded nanoscale or PSi particles by conventional aerogel synthesis routes? Does the resulting NP concentration depend upon the starting proportions in a controllable way, and can it be determined conveniently? What is the oxidation state of the PSi particles after preparation? How are the optical properties of the composite related to those of the independent materials? Here, we consider these questions.

## Methods

Metallurgical grade silicon grains of typical size around 4 microns were made porous via stain etching [8] to produce grains with a bulk silicon core and a porous silicon nanoparticle shell (we label these PSi NPs) with initially hydride-terminated surfaces. These were stored for several months in ambient conditions, and a significant amount of the surface will have oxidised, but as we shall show, they still display efficient energy transfer to oxygen, which demonstrates that some hydride-terminated surface remains [16], and we label these LH. A batch of these PSi NPs was deliberately oxidised to produce fully oxide-terminated surfaces (labelled LO); in both cases, we mean by oxidised that there is at least a monolayer of oxygen atoms back-bonded to the silicon atoms at the surface [17].

Nanoscale spherical silicon particles (Si NS) were produced in a microwave-supported plasma reactor [18] followed by etching to reduce their sizes to the point where quantum confinement effects lead to visible photoluminescence [19]; those used here had predominantly oxide-terminated surfaces (labelled SO).

Silica aerogels were prepared by a conventional one-step base-catalyst sol–gel process (described in more detail elsewhere [2]) so as to obtain a hydrophilic surface, and they were dried in supercritical CO_2_. PSi NPs or Si NSs in colloidal suspension were introduced into the mixture before the gel formation stage in concentrations from zero to 0.34 mg/ml. The potential problem of particle aggregation has also been considered for the case of Au particles [20]; we observe, however, that Si NP aggregation in ethanol is less severe than the case of Au. Blocks of aerogels were produced with dimensions of approximately 10 × 10 × 20 mm and were highly transparent if prepared without Si NPs. Aerogels containing particles of LO type were brown in colour, indicating a broad absorption spectrum in the visible range and, therefore, suggesting the presence of PSi. On the other hand, aerogels containing particles of LH type were grey black, hinting that much of the porous structure had been oxidised in preparation (the starting powder was brown coloured). Samples containing Si NSs were pale yellow to brown (with the exact colour depending on concentration), indicating again that Si NSs were present in the final composite.

Photoluminescence (PL) measurements were carried out at room temperature in vacuum, with continuous wave 325-nm excitation; low excitation densities in macro-sampling were used, and the PL was detected using a low-resolution single-grating spectrometer and CCD detector. It was also possible to cool the samples to liquid nitrogen temperatures (approximately 80 K) and to admit oxygen gas (O_2_) in order to look for the quenching of the PL by the energy transfer to adsorbed O_2_.

Raman scattering measurements were made in a macroscopic sampling mode in the back-scattering geometry using 532-nm excitation at powers low enough to avoid the well-known problems due to heating [17]. The scattered light was detected using a triple-grating spectrometer with a tunable subtractive double first stage which provided rejection of the elastically scattered light; the Raman-scattered light was dispersed in a final 1-m focal length stage with a 600-groove/mm grating and was detected using a liquid nitrogen-cooled CCD. Fourier transform infrared (FTIR) transmission measurements were made using a diamond attenuated total reflection (ATR) stage mounted on an FTIR spectrometer. The rather fragile aerogels could only be subjected to a modest mounting pressure in the ATR stage, and so the comparison of the relative strength of a given band from sample to sample was not reliable (relative peak intensities within a single spectrum are of course still meaningful). For this reason, ATR-FTIR did not provide the best means to measure any type of particle concentration; we expect that measurements of diffuse reflectance or even bulk transmission in the IR may be more appropriate, and this work is in progress.

## Results and discussion

### Photoluminescence

**Figure 1:**
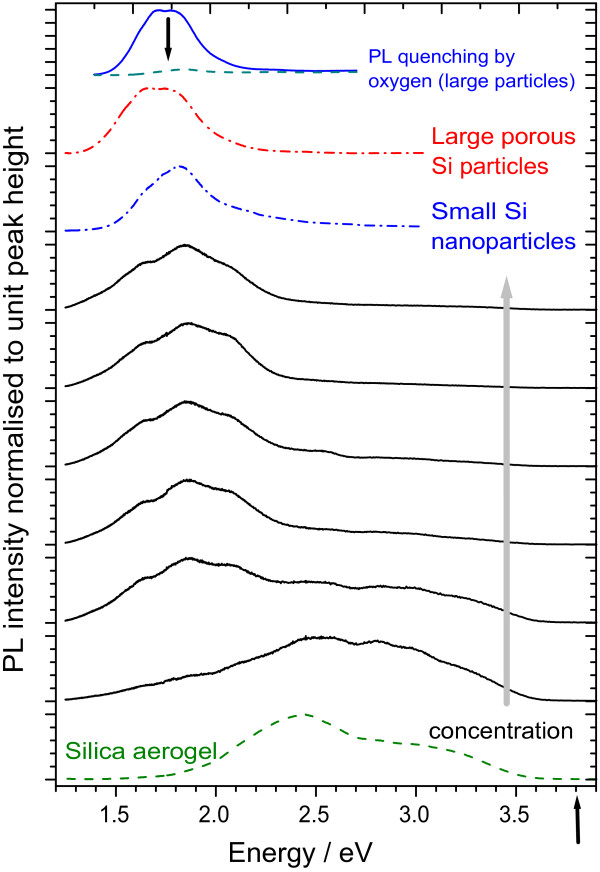
**Photoluminescence spectra of large and small Si NPs before and after incorporation into aerogels.** Photoluminescence spectra of free-standing PSi NPs of type LH (red dash-dot, second to top) and SO silicon nanospheres (blue dash-dot, third from top) and the silica aerogel without any particles (green dashed, bottom). The black, solid lines show PL spectra of aerogel-NP composites for a series of concentrations of LH particles increasing upwards (the initial concentrations in the gel preparation increase by factors of approximately 2 from 0.005 to 0.167 mg/ml). The arrow (bottom right) indicates the excitation energy of 3.81 eV (325 nm). All spectra are normalised to unit peak height. Top spectrum: un-normalised PL spectra of free-standing PSi NPs of type LH in vacuum (solid line) and in the presence of oxygen (dashed line); the arrow shows how the intensity drops on introduction of oxygen. No such change is observed for the same particles once incorporated in aerogel.

In Figure 1, we show the photoluminescence spectra of the large and small Si NPs in powder form before incorporation into the aerogels (the spectra are normalised to the PL peak height to highlight changes only in the shape of the bands). The PL spectrum of the large porous grains is dominated by silicon nanoparticles within the porous shell of each grain, so that the PL spectra of both PSi NPs and Si NSs are similar, though the PL band of the Si NSs is centred at a slightly higher energy and has a slightly lower width, suggesting a narrower size distribution for the Si NSs. When incorporated into aerogel, we see that the PL spectra of the composites are essentially those of the Si NPs still until the lowest concentrations are reached (going from top to bottom of Figure 1, which shows data for aerogels containing LH-type particles). For the lowest concentrations, the Si NP PL becomes weak in comparison to the broader, higher-energy emission from the silica aerogel itself, which is shown in the bottom PL spectrum and is typical of luminescent silica aerogels [21]. Essentially the same sequence of PL spectra is obtained also for the Si NPs of type LO in aerogel. This demonstrates that, in a first approximation, we can consider the PL of the composites as a superposition of the spectra of the Si NPs and the silica matrix; there is no evidence from this data for any interaction between the two.

At the top of Figure 1, we show also the PL spectrum of a sample of the LH particles in powder form with and without oxygen present (the arrow shows how the spectrum drops on the introduction of oxygen); this demonstrates the typical degree of quenching of the luminescence due to the energy transfer process. Detailed discussions of the evidence for this quenching mechanism have been given elsewhere [12]. By contrast, when oxygen is admitted into the aerogel samples containing Si NPs, there is no detectable change in the PL. Thus, incorporation in aerogel brings about a change in the surface state of these particles that reduces the efficiency of the energy transfer process. This is likely to be oxidation of the hydrogen-terminated surface, leading to an increased spatial separation between the confined exciton of the nanoparticle and the oxygen orbitals and, therefore, a reduction in their coupling; further work is, however, needed to quantify this. It is not yet known at which stage of the aerogel preparation this surface oxidation takes place.

### Raman scattering and FTIR

Although the colour change of the composites already indicates the state of the Si NPs after incorporation, Raman scattering is useful as a quantitative tool and because, as is usually assumed, quantum confinement and relaxation of momentum selection rules produce a shift of the frequency of the silicon Γ-point phonon mode from its bulk value of 521 cm^−1^. This shift allows one to estimate where the peak of the size distribution of the NPs lies [17,22-25]. We note that the influence of the nanocrystal surface provides an alternative physical explanation of the Raman peak shift [25], though in that model, the quantitative relationship between NP diameter and Raman shift is not altered.

**Figure 2:**
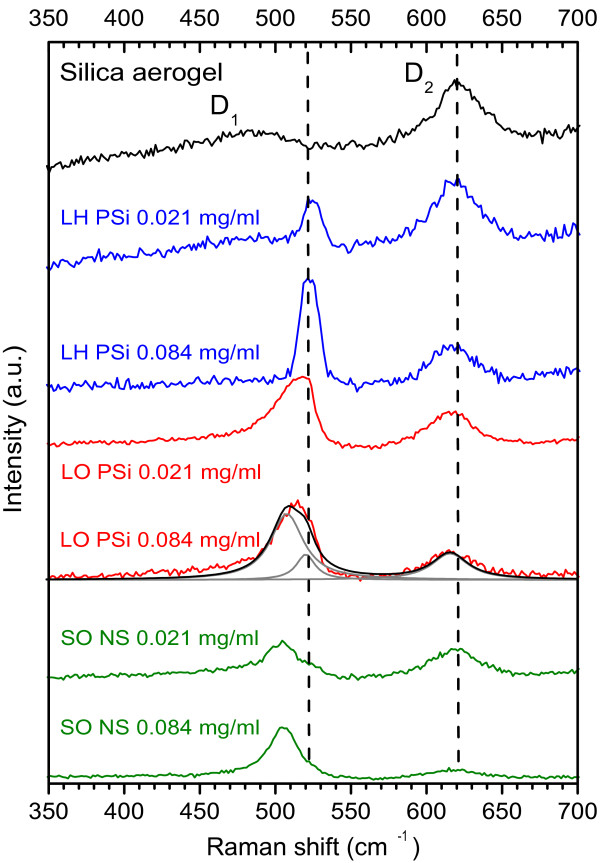
**Raman spectra of a representative selection of composite samples.** Raman spectra of silica aerogel (black, top) and aerogels containing (top to bottom) LH, LO and SO particles (blue, red and green, respectively). The vertical dashed lines indicate the Raman shifts of (left) the bulk silicon and (right) the silica D_2_ defect modes. For one of the aerogels containing LO particles, a representative fit to the data (discussed in the text) and its three components are shown (black and grey solid lines).

In Figure 2, we show the Raman spectra of a representative selection of composite samples. At the top, we show the Raman spectrum of a pure silica aerogel; the bands at 485 and 620 cm^−1^ are, respectively, the D_1_ and D_2_ defect bands of the silica matrix (their microscopic origin has been widely debated: see [1,26] and references therein). The first-order Raman scattering of the Γ-point mode of bulk silicon gives a line at 521 cm^−1^, and as Figure 2 shows, the LH aerogel composites show a line close to this position (with a Lorentzian lineshape, centre at 522 cm^−1^, FWHM 12 to 14 cm^−1^). This implies that the Raman scattering is now dominated by the remaining solid silicon core of the PSi particles and, therefore, that there is very little of the porous shell remaining (however, the PL spectra of Figure 1 do demonstrate that there are some NPs still present in the LH aerogel).

On the other hand, the aerogels containing LO particles show a Raman band that is clearly asymmetric and is shifted to lower frequency (peak at 515.5 cm^−1^, FWHM 31 cm^−1^). On the basis of these two facts and following earlier work, e.g. [22], we could attribute this to Raman scattering from Si NPs of diameter around 4 nm. However, bearing in mind the core-shell structure of these particles, we modelled the lineshape instead using a contribution at the bulk Si phonon frequency (intensity and FWHM varied but position fixed at 521 cm^−1^) and a second peak due to the nanoparticles; the fit then gives a position of 510 cm^−1^ for the second band (and FWHM 23 cm^−1^), which then implies a slightly smaller mean diameter of 2.3 nm for the nanoparticles in the remaining shell. A representative fit and its components (including the aerogel peak) are shown for one LO aerogel in Figure 2: the band arising from the porous shell is clearly dominant. Finally, the SO particles are solid and approximately spherical and so are rather analogous to Si NPs grown by ion-implantation in bulk silicon; in aerogel, we obtain a mean diameter of 2.5 to 3 nm if we interpret their Raman peaks (at 505.5 cm^−1^, FWHM 23 cm^−1^) according to the same model [22].

We now consider the concentration-dependence of the Raman scattering bands. Clearly, as the nanoparticle concentration increases and the resulting composites change from highly transmitting to opaque at the excitation energy, the penetration depth of the excitation light and the total scattering volume reduce dramatically. However, the well-defined aerogel Raman D_2_ band at 620 cm^−1^ provides a convenient means of comparing peak intensities, because it must arise from scattering within the same macroscopic volume of sample as the silicon Raman band. By taking the ratio of the integrated intensity of the silicon band (treated here, for simplicity, as a single band for any given nanoparticle type) to the aerogel D_2_ band, we should obtain a quantity proportional to the nanoparticle concentration. The validity of this normalisation depends on the structure of the silica aerogel itself not being modified by the presence of the embedded material. This may not be true; it is known, for example, that the ratio of the D_1_ to D_2_ bands gives an indication of water content in silica aerogels [26] and is modified after the UV-induced formation of CdS crystallites [1]; however, we do not expect the introduction of silicon into silica to be as dramatic a modifier of the Si-O network as water.

**Figure 3:**
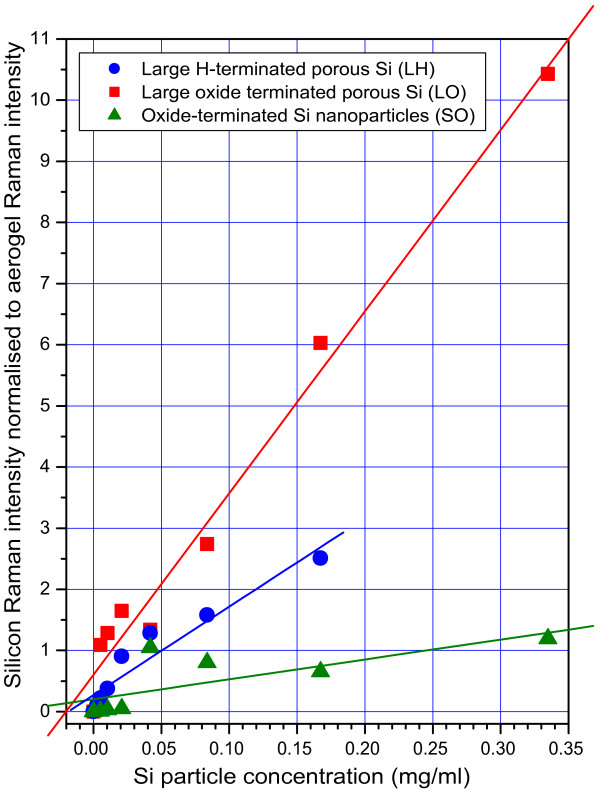
**The ratio of the silicon to silica integrated Raman intensities.** The ratio of the Raman intensities as a function of nanoparticle concentration (in the initial gel preparation) for LH, LO and SO particles (red squares, blue dots and green triangles respectively). Unconstrained linear least-squares fits to each dataset are also shown (solid lines).

Figure 3 shows a test of this idea: the ratio of the silicon Raman band to the aerogel D_2_ band is plotted as a function of the mass density of Si NPs introduced into the gel preparation, for each type of nanoparticle. Linear fits to each set of data are shown; these were not constrained to pass through the origin and do not do so exactly. It can be seen, however, that the fits converge near the origin and that the assumption of a linear relationship is reasonable. It is also apparent that the gradient of the linear fits depends on the type of nanoparticle, and this can easily be understood. Firstly, we assume that, in the case of the SO nanoparticles, a significant proportion of the particles is likely to be lost during the gel preparation process, because their mean diameter is much less than the aerogel pore size (typically 10 to 50 nm), and so they are less easily immobilised in the gel network. This accounts both for the weak degree of colouration of these composites and the low rate of increase of the Raman band with concentration. Secondly, the reduction in Raman strength of about a factor of 2 between the LO particles and the LH particles (for the same initial concentration) is qualitatively consistent with our earlier conclusion that the porous shell of the LH particles becomes oxidised, leading to a loss of scattering cross-section of the silicon phonon mode. Of course, the fundamental Raman scattering cross-section for these different types of particle is not necessarily identical, and any variation of this will also lead to a difference in slope of the fits in Figure 3. To estimate the importance of this effect, we plan to investigate the dependence of the Raman spectra on excitation energy.

**Figure 4:**
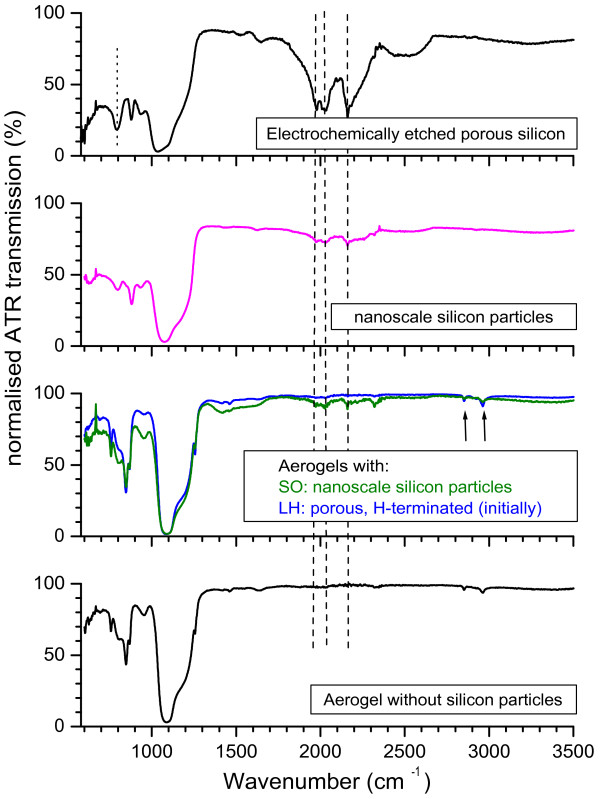
**Fourier transform infrared transmission spectra in the attenuated total reflection configuration.** Partially oxidised porous silicon (top), free SO nanoparticles (magenta), aerogels containing nanoparticles of SO (green) and LH (blue) types, and pure silica aerogels (bottom). The spectra are all baseline corrected and normalised to 3% transmission at the Si-O-Si absorption band at approximately 1,100 cm^−1^ so as to show the relative strength of the Si-H modes indicated by the vertical dashed lines. The peaks indicated by arrows in the aerogel spectra are due to CH_3_ absorptions.

Finally, we have tested the above conclusions using FTIR, since this provides a sensitive identification of Si-O-Si and Si-H vibrational modes via their IR absorption bands at around 1,030 and 1,180 cm^−1^ (Si-O-Si) and 2,000 to 2,200 cm^−1^ (Si-H, Si-H_2_ and Si-H_3_ stretches) [9,27]. On Figure 4, the vertical dashed lines show the positions of the bands associated with the three Si-H stretching modes. These modes are readily seen in, for example, conventional electrochemically etched PSi (top) even after it is several months old and substantially oxidised (which is indicated, for instance, by the presence of the band at 790 cm^−1^ marked by the dotted line [9]). The Si-H modes are also weakly present in the IR spectra of the nanoscale silicon particles before and even after aerogel preparation (second from top) despite the fact that they are mostly oxidised. The FTIR spectra of the composite aerogels made using LH particles, however, do not show any sign of these bands, and we obtain spectra that are dominated by the IR absorption of silica aerogel (shown at the bottom of Figure 4 for comparison) even for samples that contain sufficient concentrations of nanoparticles that they are opaque for visible light. The silica aerogel IR spectra show the expected Si-O modes but also two weak modes arising from Si-CH_3_ groups indicated by the two arrows [28]. The fact that these are only weak is consistent with the aerogel surface being hydrophilic (a large Si-CH_3_ coverage leads to a hydrophobic surface). Finally, as one would expect, we see no sign of Si-H modes in the aerogels containing large, oxidised particles, and their FTIR spectra (not shown in Figure 4) are very similar to those of our silica aerogels and those reported in the past [28]; we note that the Si-Si modes of the bulk silicon cores will not be detected via IR absorption.

## Conclusions

We have demonstrated that silicon-silica aerogel composites can be produced by conventional aerogel synthesis with the addition of either nanoscale silicon particles or macroscopic porous silicon grains. Substantial oxidation of the silicon takes place if its surface is partially hydride terminated, and this can be severe enough to remove a large fraction of the porous silicon structure, though a solid crystalline silicon core remains. For oxide-terminated porous silicon grains, however, more of the porous silicon structure survives, though it is (as expected) not active in energy transfer to adsorbed oxygen.

## Competing interests

The authors declare that they have no competing interests.

## Authors’ contributions

JA carried out the luminescence and Raman measurements. DW proposed the Raman measurements, contributed to the analysis of the Raman data and carried out the FTIR measurements. BG and SP prepared the Si nanoparticles. DK conceived of the search for energy transfer to oxygen. MG and MR prepared the aerogels, and TAB proposed the use of aerogels for photonic and sensing applications of this type. All authors contributed to planning this work and read and approved the final manuscript.

## Authors’ information

JA, BG, SP, MR and MDWG were postgraduate research students at the University of Bath during this work; DW is a senior lecturer in Physics at the University of Bath with a special interest in optical spectroscopy, and TAB and DK are professors of Physics at the University of Bath with interests in photonics and porous silicon, respectively.
